# Atlas of Interactions Between Decoration Proteins and Major Capsid Proteins of Coliphage N4

**DOI:** 10.3390/v17010019

**Published:** 2024-12-26

**Authors:** Klem McJarrow-Keller, Alice-Roza Eruera, Alexander J. M. Crowe, Rosheny Kumaran, Jaekyung Hyun, Mihnea Bostina

**Affiliations:** 1 Department of Microbiology and Immunology, University of Otago, Dunedin 9016, New Zealand; mcjkl477@student.otago.ac.nz (K.M.-K.); alice.eruera@otago.ac.nz (A.-R.E.); croal939@student.otago.ac.nz (A.J.M.C.); kuros488@student.otago.ac.nz (R.K.); 2 School of Pharmacy, Sungkyunkwan University, Suwon 16419, Republic of Korea; jhyu002@gmail.com

**Keywords:** cryo-electron microscopy, bacteriophage N4, capsid decoration protein, Ig-like decoration protein

## Abstract

Coliphage N4 is a representative species of the *Schitoviridae* family of bacteriophages. Originally structurally studied in 2008, the capsid structure was solved to 14 Å to reveal an interesting arrangement of Ig-like decoration proteins across the surface of the capsid. Herein, we present a high-resolution N4 structure, reporting a 2.45 Å map of the capsid obtained via single particle cryogenic-electron microscopy. Structural analysis of the major capsid proteins (MCPs) and decoration proteins (gp56 and gp17) of phage N4 reveals a pattern of interactions across the capsid that are mediated by structurally homologous domains of gp17. In this study, an analysis of the complex interface contacts allows us to confirm that the gp17 Ig-like decoration proteins of N4 are likely employed by the virus to increase the capsid’s structural integrity.

## 1. Introduction

The study of phages has undergone major developments in recent decades, particularly with regard to their application in molecular biology [1,2,3]. Phages outnumber bacteria tenfold, shaping bacterial evolution and displaying vast structural and genetic diversity [4,5]. The most commonly isolated and well-studied group of phages is the order *Caudoviricetes*, or tailed phages [6]. These phages have an icosahedron-based head containing the dsDNA genome, formed from copies of a major capsid protein (MCP) that adopts the HK97-like fold [7]. One vertex is replaced with a variable tail apparatus, which adsorbs onto the exterior surface of the host cell and forms the conduit to carry the viral genome across the cell wall and membrane and into the host cytoplasm [7]. Often, these capsids have exterior ‘decoration’ proteins that stabilise the capsid and/or perform other functions, such as host adhesion or bacterial hitchhiking [8].

Phage N4, isolated in 1965, is a lytic phage that infects *Escherichia coli* K12 strain, with a short, non-contractile tail characteristic of podophages and a 70 kb dsDNA genome (NCBI accession EF056009) [9,10]. N4 persisted as a genetic orphan until the discovery of two further N4-like viruses in 2009 [11], with discoveries of more N4-like phages leading to the establishment of a new family, *Schitoviridae*, in 2021 [10,12]. As N4 was investigated, it revealed unique features that have motivated continued research into the virus and its relatives. These include a delayed host lysis and large burst size [9], terminal microheterogeneity in its genome [13] and the use of three RNA polymerases, including a large one (~3500 amino acids) packaged inside the capsid [14,15].

Despite ongoing research into the genetics and biochemistry of *Schitoviridae*, limited structural information exists on the family. In 2008, the capsid and tail components of N4 were identified using cryogenic-electron microscopy (cryo-EM) and mass spectrometry [15]. The capsid, resolved to 14 Å resolution (EMDB-1472), adopts the triangulation number T = 9, corresponding to 535 copies of the MCP gp56. The capsid interfaces with the tail via a neck (unidentified) and portal (gp59) assembly. On top of the tail column, internal core protein density was observed within the capsid. This was putatively assigned to the ejectosome, a structure ejected into the host cell wall to form the periplasmic tunnel that allows for the safe passage of viral DNA into the host [16,17]. On the surface of the capsid, three copies of a decoration protein, gp17, were observed per the asymmetric unit (ASU). Based on the unpublished experiments regarding the capsid stability of mutants lacking gp17 and sequence analysis, gp17 was predicted to play a cementing role and to consist of three immunoglobin-like (Ig-like) domains.

The Ig-like domain, characterised by a beta-sandwich fold, is found in various phage proteins, including the MCP, as in the case of phage phi29 [18], tail sheath proteins [19] or decoration proteins of tailed phages. Examples of the latter include the highly antigenic outer capsid protein (Hoc) of T4 [20,21], pb10 of T5 [22] and the vertex decorations of *Vibrio* phage SIO-2 [23]. These Ig-like decoration proteins are expected to be non-structural [8], with the exceptions of pb10 [22] and potentially gp17 of N4 [15]. Gp17 of N4 is the only published phage capsid decoration with multiple Ig-like domains that lie flat across the capsid surface rather than protruding outwards from it [15,23]. Hoc from T4, for instance, consists of three Ig-like domains attached to a C-terminal capsid-binding domain, forming long fibres that project from the capsid surface and are thought to be involved in host attachment and regulating virion aggregation [20].

Here, we update the structural knowledge of N4, reporting a 2.45 Å map of the capsid using single particle cryo-EM. The improved resolution enabled the modelling of the constituent MCP and decoration proteins (gp56 and gp17). Thereafter, we analysed the complex interface contacts made between the capsid components, providing insight into the structural properties of N4-like capsids and, through gp17, further exploring the diversity of Ig-like decorations in *Caudovirales*.

## 2. Materials and Methods

### 2.1. Virus Production and Purification

*Escherichia coli* (*E. coli*) phage N4 was produced and purified using adapted methods [15]. Briefly, 2 mL of overnight *E. coli* K12 1655 ΔRM bacterial culture was inoculated into 50 mL of Luria–Burtani (LB) broth supplemented with 5 mM CaCl_2_ and 20 mM MgSO_4_, outgrown for 1.5 h at 37 °C and then infected with 5 µL of phage stock lysate. The sample was incubated overnight, and the lysate was collected the following day. A second passage was performed to bulk the phage titer to a final concentration of 3.2 × 10^11^ PFU/mL, determined by plaque assay. The lysate was then layered onto 20% sucrose (*w*/*v*) and centrifuged at 50,000× *g* for 1.5 h at room temp in a Beckman Coulter ultracentrifuge. The pellets were resuspended overnight in phage buffer (10 mM Tris–HCl pH 7.4, 10 mM MgSO4 and 0.01% *w*/*v* gelatin) and assessed by negative stain EM for sample purity (Supplementary Figure S1), stained using 1% phosphotungstic acid and imaged on a JEOL 1400 Flash TEM fitted with an Oxford EDS system.

### 2.2. Data Collection and Processing

Briefly, 5 µL of purified N4 phage was vitrified using an FEI Vitrobot IV on a Quantifoil R1.2/1.3 Au200 with 2 nm C-film grid. Data were collected on a TFS Krios G4 operating at 300 KeV with a K3 BioContinuum direct electron detector (AMETEK) operating in electron counting and super-resolution. Exposures were taken using EPU (Thermo Fisher Scientific, Waltham, MA, USA) at a calibrated magnification of ×60,901 with a total dose of 53.59 e/Å2 fractionated over 50 frames (Supplementary Table S1). Data were processed in cryoSPARC v4.5.3 with 69 optics groups enabled to correct the parameters used for data collection [24]. Particles were blob-picked, sorted by iterative 2D classification and submitted to ab initio reconstruction to produce an initial volume for downstream refinement. Next, the volume was submitted for homogeneous refinement followed by non-uniform refinement with negative Ewald sphere correction enabled. A total of 157,663 particles were used in the final capsid reconstruction, with a final map resolution of 2.45 Å, according to the golden standard Fourier shell correlation with the 0.143 threshold.

### 2.3. Modelling and Data Analysis

Initial models were produced using AlphaFold3 and refined into the map with ISOLDE [25,26]. Manual refinement was done in Coot v.0.9.5. Geometries were corrected in ISOLDE, and the models were automatically refined in Phenix v.1.19.1 [27,28,29]. A table of collection and refinement statistics is reported in Supplementary Tables S1 and S2. Root mean square deviation of atomic positions (RMSD), inter-chain contacts and atomic distances were measured in UCSF ChimeraX v1.8 [30], and the buried surface areas between the chains were calculated in PISA [31]. The angular difference in orientations of gp17 DIII was determined by comparing the orientations of each domain’s axis, as defined in UCSF ChimeraX, after aligning the DII structures. The quality of final reconstructions was assessed using the Molprobity score, Q score and EMRinger score [32,33]. The views of the relative map-model fits and orthogonal slices are reported in Supplementary Figures S2, S4 and S5, according to the best practice community guidelines [34]. Sequence alignments were performed using the NCBI Constraint-based Multiple Alignment Tool (COBALT) and BLAST [35,36].

## 3. Results and Discussions

### 3.1. Overall Structure of Coliphage N4 Capsid

**Figure 1:**
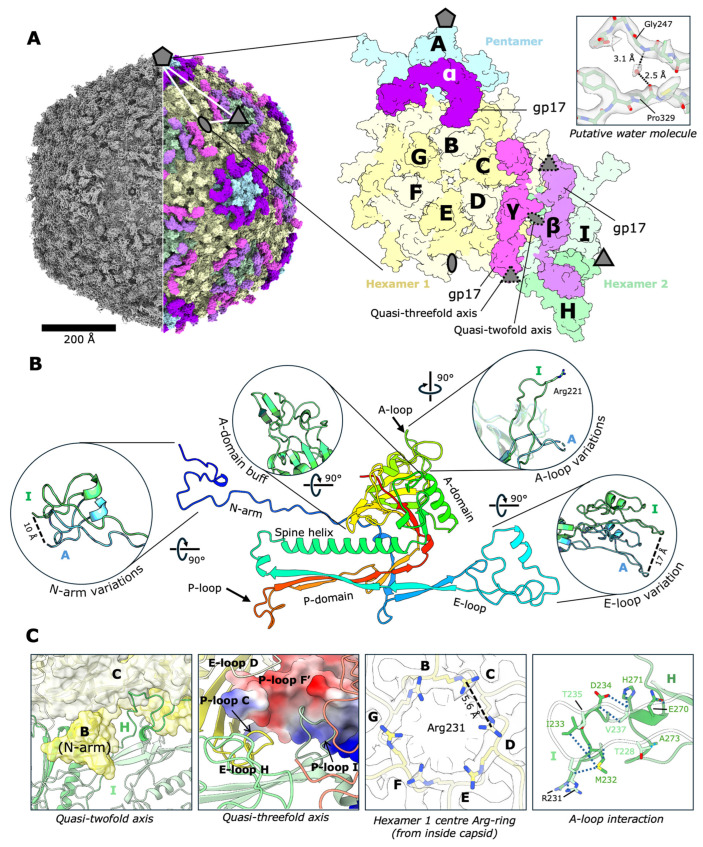
The arrangement and variation of MCPs in the N4 capsid. (**A**) The cryo-EM density map (left, contour level 0.239) is compared to the atomic model of the N4 capsid with a smoothed van der Waals surface displayed (calculated in UCSF ChimeraX v1.8). The pentameric MCPs are coloured blue, and the hexameric MCPs are coloured green and yellow. The gp17 decoration proteins are coloured in shades of pink, according to their relative position on the capsid. The density for a putative water molecule in chain I is shown with the distance measured in UCSF ChimeraX (top panel, contour level 0.725). (**B**) Richardson diagram of an MCP coloured from N- (blue) to C-terminus (red). The domains of interest are highlighted and compared between chains A and I in green and blue, respectively. (**C**) Selected MCP interfaces were analysed in UCSF ChimeraX, and contact side chains involved in interactions are displayed where relevant. Arg-ring density is displayed at contour level 0.286. P-loop F’ refers to the P-loop of chain F from the adjacent ASU.

The N4 capsid was solved using cryo-EM to a final resolution of 2.45 Å (Figure 1). The map showed good isotropic density for most side chains, and in the highest resolution areas (determined by local resolution estimation), some uniform densities can be observed, which we assign to be putative water molecules (Figure 1A). The map confirms the previous conclusions with respect to the gross structure of the N4 capsid, with a T = 9 geometry of MCPs (gp56) (Supplementary Figures S8 and S9), and three copies of the decoration protein gp17 per ASU. The N-terminal arm of the MCP (residues 1–46) extends to the quasi-2-fold axis of clockwise adjacent MCPs. Similar to that of phage P74-26 [37], an extended P-domain loop (residues 358–373) forms the adjacent quasi-3-fold axis, forming an inter-capsomeric interface at the nearby MCP’s P-domain. The E-loop also features an insertion that widens the contact area with the adjacent P-domain within the capsomer and is part of the decoration protein interface.

**Figure 2:**
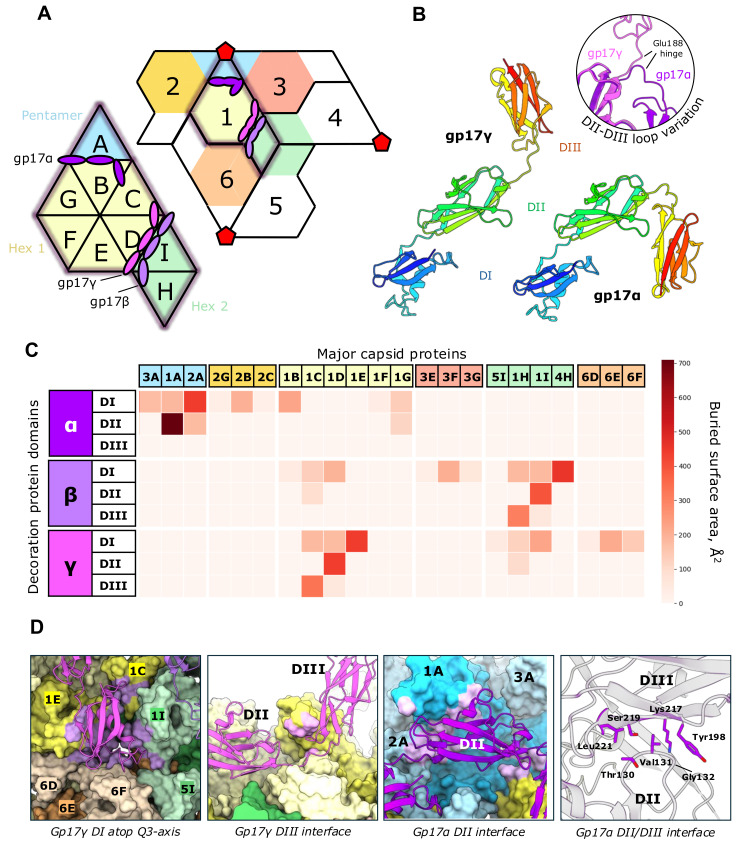
Decoration interactions with MCPs. (**A**) ASU and relative capsid surface map numbering convention (**B**) Richardson diagrams of decoration proteins gp17 coloured from N- (blue) to C-terminus (red). Gp17β is shown as a reciprocal of gp17γ. An overlay of the DII-DIII loop between gp17γ and gp17α is shown in the right panel. (**C**) A heatmap of buried surface area (Å2) is calculated in PISA against each decoration protein and each MCP. (reference for PISA) (**D**) A selection of gp17 interfaces. Highlighted MCP surfaces indicate decoration-contacting residues.

A feature unique to the MCP of the N4 and the newly described DEV phage [38] is an additional loop (residues 223–242), which lies along the inter-A-domain interfaces in the centre of the hexamers but is disordered within the pentameric MCPs (Figure 1B,C and Supplementary Figure S10). Within the quasi-6-fold axes at the centres of hexamers, six arginine sidechains (one from each MCP) extend from the A-loop into the centre with an average distance of 5.7 Å between the adjacent neighbours (see Figure 1C). The A-domain also contains two extended loops (spanning residues 184–203, 300–328), forming a protrusion on the outer surface of the capsid, which serves to direct the orientation of the domain DIII of the gp17 chains β and γ (Figure 2D). However, phage DEV lacks decorations and is only distantly related to N4, suggesting these MCP characteristics may be common across *Schitoviridae* and have functions unrelated to the presence of Ig-like decorations.

### 3.2. Ig-like gp17 Decoration Proteins

The three unique positions of gp17, hereafter referred to as gp17α, gp17β and gp17γ, were identified to bind around quasi-2-fold axes of symmetry. Gp17 forms three beta-strand-rich Ig-like domains, labelled DI-DIII, from the N-terminus to the C-terminus, separated by a flexible linker. DI and DII were of sufficient resolution for atomic modelling of most sidechains and all of the carbon backbone (Supplementary Figure S3). However, DIII extends into the solvent-exposed region and is significantly flexible (Figure 2B). To allow for analysis, DIII was replaced with a rigid-body fit AlphaFold3 model (Supplementary Figure S3). DALI searches confirm the Ig-like topology of gp17 (Supplementary Figure S7).

Although Ig-like decoration proteins are common, they typically extend perpendicular from the capsid surface, as observed in phage tentaclins and T4 Hoc [20,39]. Interestingly, coliphage N4 decorations bind horizontally to the MCP–MCP interfaces. A copy of the DI domain sits on each quasi-3-fold axis, with DII lying principally on the P-domain of a particular MCP (Figure 1A and Figure 2A). The DIII domain differs considerably in orientation between gp17α and the other two chains due to a bend in the DII–DIII linker by about 130°. Gp17β and gp17γ are nearly structurally identical, lying along either side of a single quasi-2-fold axis dividing the two distinct hexamers.

Our ‘interaction atlas’ (Figure 2C) describes the interfaces made by gp17 with the capsid surface. A comparison among different capsomers reveals that the decorations form certain interactions very similarly, such as with the capsomer on the other side of the quasi-2-fold axis adjacent to each DII (1F, G, B for gp17α; 1B–D for β; 5I–1I for γ, see Figure 2C). In contrast, some interactions are not common between domains; for instance, the DIII domain in gp17β and gp17γ interacts weakly with the capsid, while the gp17α DIII does not. These differences are dictated by different pseudo-two-fold environments provided at the pentamer–hexamer and hexamer–hexamer interfaces.

Comparing the amino acid sequences of DI, DII and DIII to each other reveals significant dissimilarity in the amino acid sequence between each domain despite relatively high structural conservation (Supplementary Figure S6). Although we used an icosahedral reconstruction, we believe that the interactions described in Figure 2C are present in the full N4 virion, as shown in the low-resolution reconstruction reported previously [15].

Phage decoration proteins have been implemented in host recognition or initial attachment, bacterial hitchhiking or, in many cases, capsid reinforcement [8]. The capsid reinforcement benefits phage fitness by helping to resist the internal pressures exerted by the genome and packaged proteins, such as the ejectosome and large RNA polymerases [8,15,40]. The N4 decoration displays an unusual binding arrangement. Chain α binds the pentamers in the region of the highest curvature, while chains β and γ bind about just one of two unique quasi-2-fold axes near the three-fold axis at the sites of lowest curvature. This could be explained by the strong interaction of DII from chain α with the pentamers when compared with other chains (see Figure 2C), which shows a preference for the pentamer binding sites. We propose that a consistent binding pattern benefits the capsid stability by ensuring a copy of gp17 DI is present to reinforce every quasi-3-fold axis of the capsid. The minimal capsid contact formed by DIII implies that this domain has an uncertain, non-structural function that seems to be similar to Ig-like domains observed in other phages. Future research could explore the impact of mutagenesis on gp17, perhaps by progressively truncating domains from gp17, to confirm the role of each domain in the N4 virion.

## 4. Conclusions

The cryo-EM structure of the coliphage phage N4 updates our current understanding of the Ig-like decoration proteins in reinforcing the capsid. The structures of these decoration proteins reveal an unusual supine arrangement around quasi-2-fold axes that contribute to capsid structural resilience, particularly via key stabilising contacts at quasi-3-fold axes, and a possible accessory function for the third domain of gp17.

## Data Availability

Data were deposited according to community best practices [34]. The models and associated maps, including half maps and solvent masks, were aligned and deposited to the PDB and EMDB under accession codes 9E99 and EMD-47777, respectively.

## Supplementary Materials

The following supporting information can be downloaded at: https://www.mdpi.com/article/10.3390/v17010019/s1. Table S1: Data collection statistics. Table S2: Reconstruction and refinement statistics. Figure S1: Negative stain images of Coliphage N4. Figure S2: Plots associated with the cryoSPARC non-uniform refinement of the N4 capsid. Figure S3: Modelled and unmodelled regions. Figure S4: Map-to-model fit of MCPs in N4 capsid cryo-EM density map [34]. Figure S5: Map-model fit of decoration 1. Figure S6: N4 decoration protein secondary structure [35,41]. Figure S7: Structural comparison of IG-like domains via DALI search. Figure S8: MCP chains in context of capsomers. Figure S9: MCP topology diagram [41]. Figure S10: Comparison of the DEV and N4 major capsid proteins [35].
