# Supplementary material for: Atlas of Interactions Between Decoration Proteins and Major Capsid Proteins of Coliphage N4
Source: Viruses. 2024 Dec 26;17(1):19. doi: 10.3390/v17010019 (PMC11768535; doi:10.3390/v17010019)
Supplement: Supplementary file 1 [file viruses-17-00019-s001.zip › viruses-3360660 - supplementary.pdf]

viruses MDPI

Supplementary Materials

Supplementary Table S1. Data collection statistics.

Sample: Bateriophage N4
Date: 2024-04-26
Operator: Jaekyung Hyun

Grid prep.	EM-Grid	Quantifoil R1.2/1.3 Au200 with 2nm C-film
	Glow discharge device	Solarus II (Gatan)
	Glow discharge	Negative
	Forward RF target	20W
	Forward RF range	5W
	Gas flow	Argon 30sccm
	Vacuum	70mTorr
	Additional step	poly-D-lysine treatment on discharged grid
Vitrification	Loading volume	3.5ul
	Loading side	carbon
	Blot time	5sec
	Blot force	5
	Wait time	1-2 minutes
	Temp/hum	4°C/90%
Microscopy	Model	Krios G4
	Spherical abberation, Cs	2.7mm
	Dose rate	10e/pix/sec
	Pixel size	0.821Å/pix (super-resolution)
	Nominal magnification	X53,000
	Calibrated magnification	X60,901
	Exposure time	14.45sec
	# of fractions	50
	Condenser apersure	70um
	Objective lense aperture	N/A
	Total dose	53.59e/Å^2
	Dose per fraction	1.07e/Å^2
	AFIS	Yes (68 optics groups)
	Alpha tilt	none
Data	Model	Gatan K3 BioContinuum
	Energy filter slit width	20eV
	Acquisition mode	EC / super-resolution
	Correlated double sampling (CDS) mode	Used
	Movie format	tiff(LZW)
	Gain normalization	no
	Gain rotation of flip (CryoSparc)	Y-flip required
	Gain rotation of flip (Relion)	unknown

Supplementary Table S2. Reconstruction and refinement statistics.

	MCP A	MCP I	gp17α
**Data collection**			
Microscope	TFS Krios G4		
Voltage	300 kV		
Target defocus range (μm)	0.5 - 1.5		
Pixel size	1.0673 Å		
Average e^- dose per fraction	1.07 e^-/Å^2		
Particles (initial)	150,000+		
Particles (final (%))	112,193 ()		
**Reconstruction**			
Symmetry	I2		
Resolution (unmasked)	3.0 Å		
Resolution (masked)	2.45 Å		
Map-sharpening B-factor	-86.4 Å^2		
**Model composition**			
Non-hydrogen atoms	3028	3090	3430
Protein residues	393	401	278
**RMS Deviations**			
Bonds (Å)	0.004	0.004	0.016
Angles (°)	0.948	0.969	2.029
**Validation**			
Clashscore	1.66	0.49	13.51
Rotamer outliers (%)	0.31	0.30	3.07
Ramachandran plot favoured (%)	98.20	98.25	97.10
Ramachandran plot outliers (%)	0	0	1

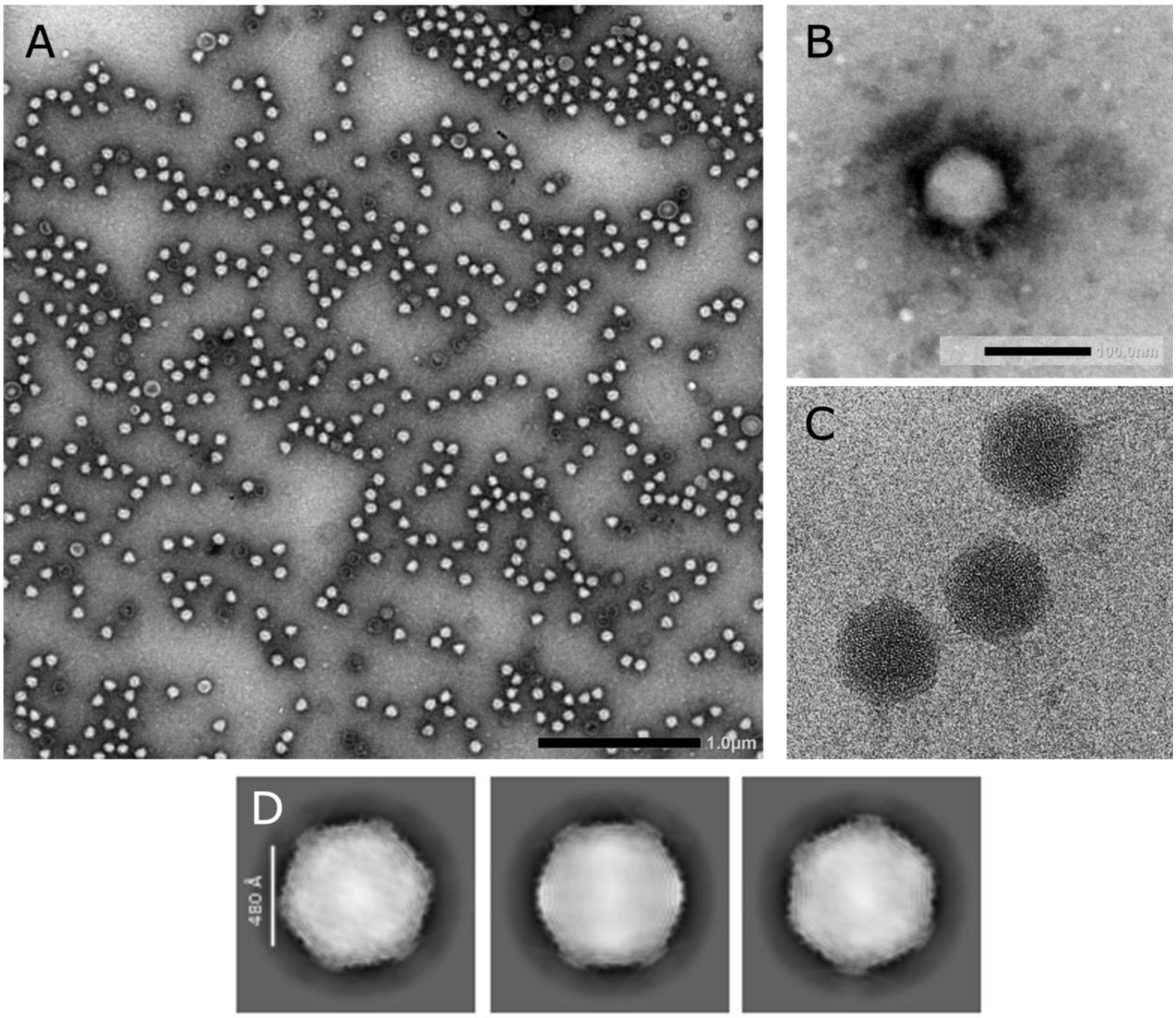

Supplementary Figure S1. **Negative stain images of Coliphage N4.** (**A**, **B**) C-flat grids were plasma discharged in a GloQube Plus (Quorum Technologies) and loaded with 10 uL of N4 phage sample at 1 x 10^{13} PFU/mL. The grid was stained with 1% PTA and imaged in a JEOL Flash 1400 TEM. Capsids have a diameter ~80 nm and tail dimensions ~30-40 nm length from the neck. (**C**) Cryo-EM micrograph image of N4. (**D**) 2D class averages of the N4 capsid.

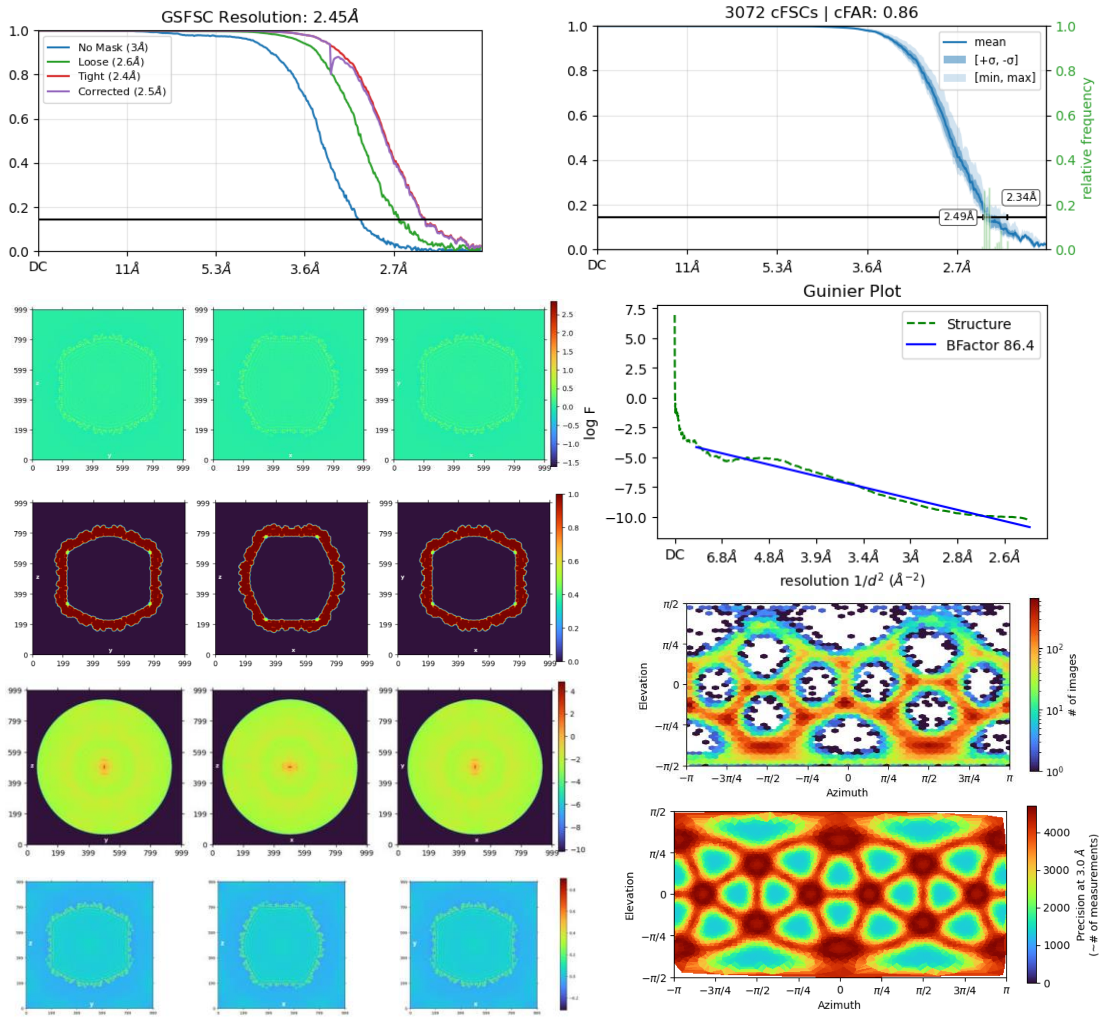

Supplementary Figure S2. Plots associated with the cryoSPARC non-uniform refinement of the N4 capsid. Gold-standard Fourier Shell Correlation plots, Guinier (B factor) plot, real space, Fourier space and mask slices across three orthogonal directions (x, y, z), and viewing direction distribution plots are provided.

MCP a, b, c, d, e, f, g, h, i	100% coverage : residues M1 – L401
Decoration α*	Domain I Domain II *Domain III
Decoration β*	*Domain III
Decoration γ*	*Domain III

*Due to lack of continuous density for domain III, AlphaFold3 prediction of Domain III was rigid-body fit into the map.

Supplementary Figure S3. Modelled and unmodelled regions. Regions for each protein model which were unmodelled due to a lack of clear, well-defined density are indicated by coloured bar.

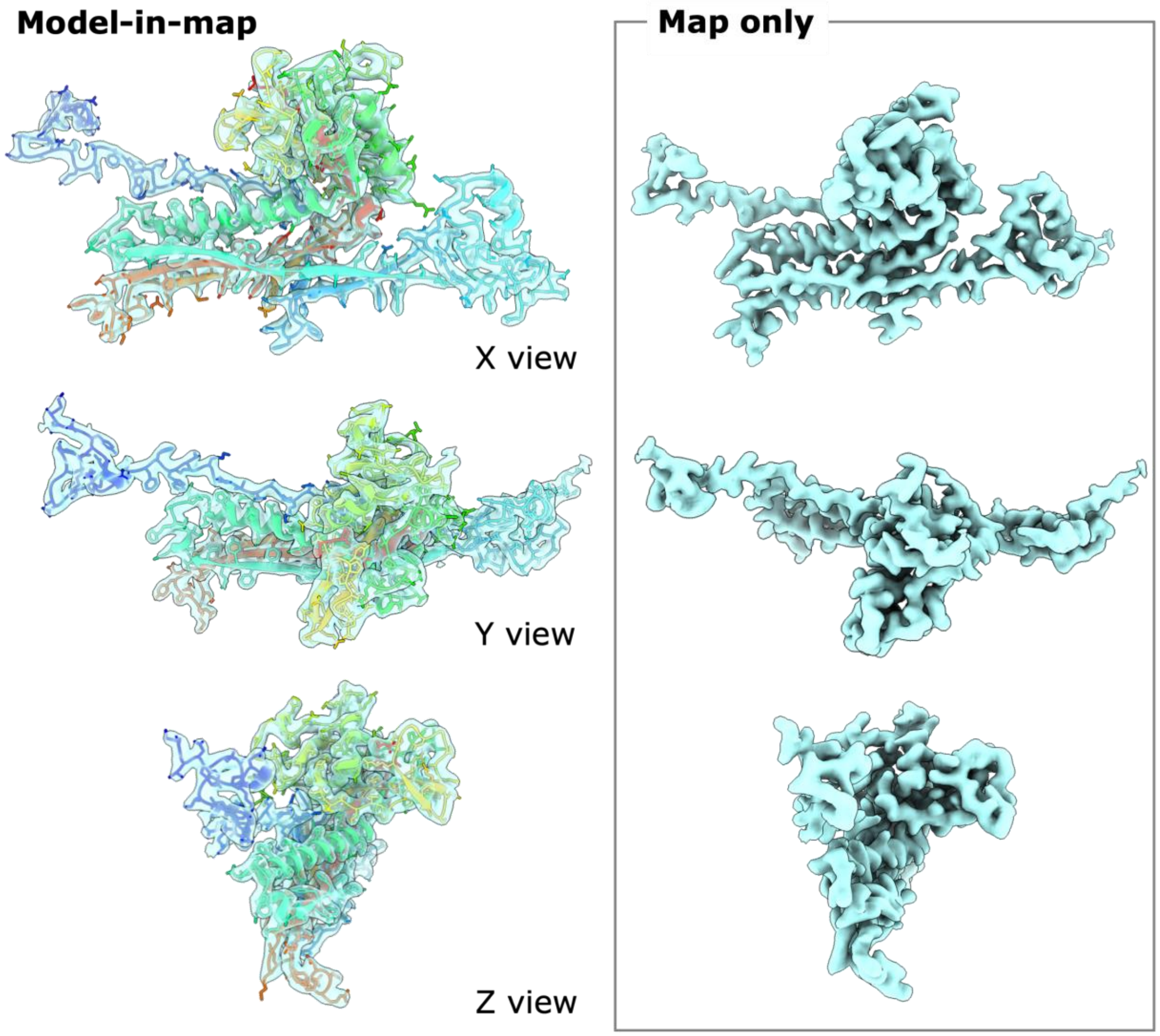

Supplementary Figure S4. Map-to-model fit of MCPs in N4 capsid cryo-EM density map. The map-to-model fit of MCP chain A is shown from a series of orthogonal directions according to the recent best practice community guidelines [34]. The map is displayed with map contoured to level 0.14 visualised on UCSF ChimeraX.

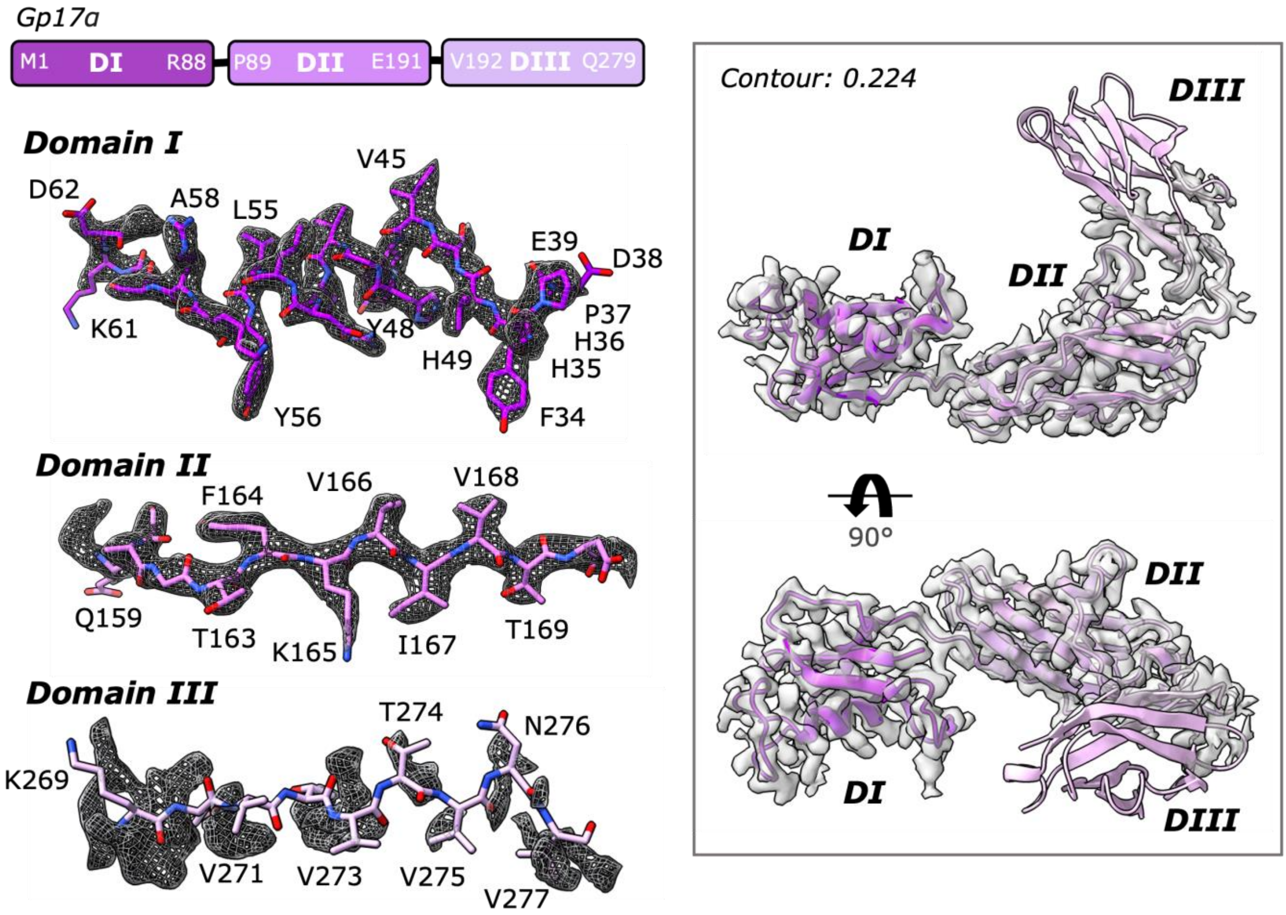

Supplementary Figure S5. Map-model fit of decoration 1. All maps were smoothed and sharpened for display in UCSF ChimeraX using commands '*surfaceSmoothing true smoothingIterations 4 subdivideSurface true*'. Residues from Domain I (Tyr41 to Asp62) are displayed inside a zoned map contoured at level 0.418 and Domain II (Glu159 to Asp170) is displayed with the map contoured to level 0.391. Domain III (Lys269 to Val277) is displayed contoured at 0.222.

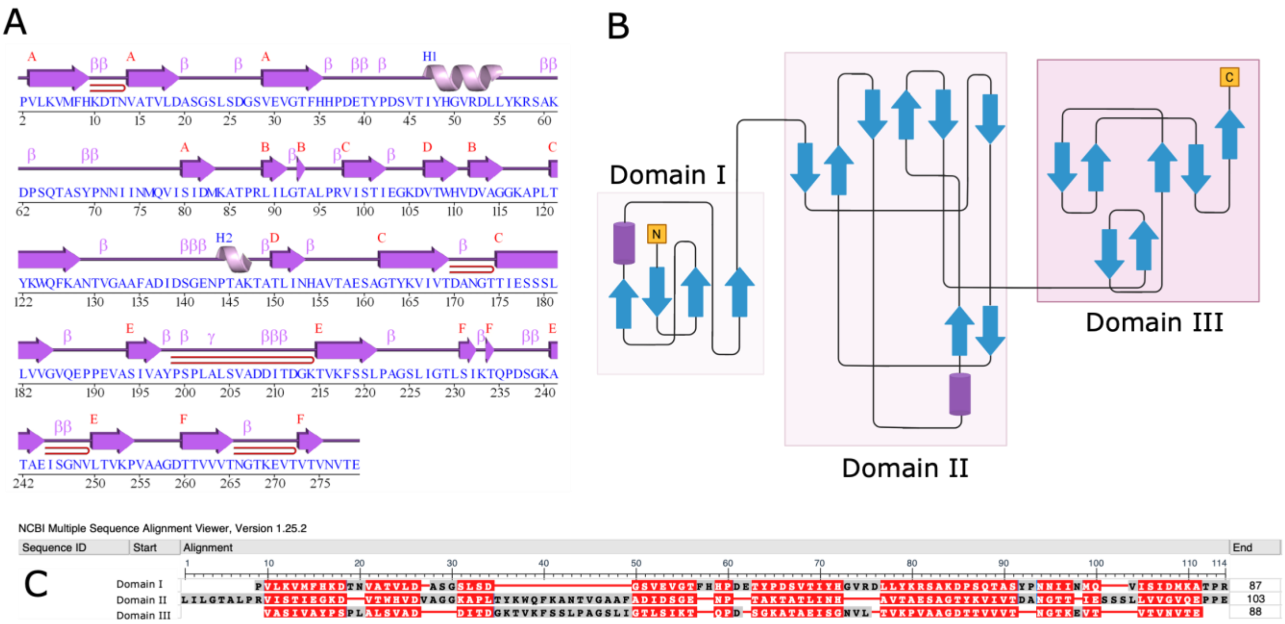

Supplementary Figure S6. N4 decoration protein secondary structure. (**A**) A 2D topology diagram was generated through the PDBSum server [41]. (**B**) The domain-based topology plot from PDBSum was replotted manually for ease of visual interpretability. (**C**) Sequence alignments were performed by submitting individual domains to the NCBI Constraint-based Multiple Alignment Tool (COBALT) [35].

DALI results against gp17 amino acid sequence

Summary

No:	Chain	Z	rmsd	lali	nres	%id	PDB	Description
1:	3k0w-A	12.6	8.7	104	184	12	PDB	MOLECULE: MUCOSA-ASSOCIATED LYMPHOID TISSUE LYMPHOMA
2:	7y9a-A	12.2	4.1	107	192	13	PDB	MOLECULE: DOWN SYNDROME CELL ADHESION MOLECULES;
3:	3puc-A	12.1	2.1	92	98	17	PDB	MOLECULE: TITIN;
4:	3laf-A	12.1	9.4	125	384	22	PDB	MOLECULE: DELETED IN COLORECTAL CANCER;
5:	3b43-A	12.1	8.6	134	569	19	PDB	MOLECULE: TITIN;
6:	2iep-A	12.0	9.7	110	187	15	PDB	MOLECULE: MUSCLE-SPECIFIC KINASE RECEPTOR;
7:	3jxa-B	12.0	3.2	106	383	17	PDB	MOLECULE: CONTACTIN 4;
8:	3dmk-A	12.0	5.3	125	775	11	PDB	MOLECULE: DOWN SYNDROME CELL ADHESION MOLECULE (DSCAM) ISOF
9:	3uto-B	11.9	3.2	105	566	14	PDB	MOLECULE: TWITCHIN;
10:	8ht3-A	11.8	2.2	93	104	15	PDB	MOLECULE: NEW ANTIGEN RECEPTOR VARIABLE DOMAIN;
11:	1g1c-A	11.8	1.9	91	98	16	PDB	MOLECULE: IMMUNOGLOBULIN-LIKE DOMAIN I1 FROM TITIN;
12:	7ahs-B	11.8	2.8	95	272	16	PDB	MOLECULE: ISOFORM 5 OF TITIN;
13:	5i99-A	11.7	12.0	118	398	14	PDB	MOLECULE: CONTACTIN-3;
14:	1u2h-A	11.7	1.9	89	96	18	PDB	MOLECULE: AORTIC PREFERENTIALLY EXPRESSED PROTEIN 1;
15:	5k6w-A	11.7	14.4	122	475	10	PDB	MOLECULE: PROTEIN SIDEKICK-1;
16:	6fwx-B	11.6	3.3	128	195	20	PDB	MOLECULE: TITIN,TITIN,TITIN;
17:	5oyj-C	11.6	12.3	117	223	19	PDB	MOLECULE: DARPIN D4B;
18:	3qp3-A	11.6	2.6	94	101	13	PDB	MOLECULE: TITIN;
19:	7s83-A	11.6	2.1	94	114	18	PDB	MOLECULE: SHAB02 VNAR;
20:	9ba4-A	11.5	15.4	133	970	16	PDB	MOLECULE: CONTACTIN-2;
21:	8p35-A	11.5	2.0	94	100	16	PDB	MOLECULE: TITIN;
22:	4u7m-A	11.5	10.5	109	289	19	PDB	MOLECULE: LEUCINE-RICH REPEATS AND IMMUNOGLOBULIN-LIKE DOMA
23:	1tlk-A	11.5	4.0	96	103	11	PDB	MOLECULE: TELOKIN;
24:	2nzi-A	11.5	11.0	110	292	20	PDB	MOLECULE: TITIN;
25:	7y6o-A	11.4	8.2	124	287	12	PDB	MOLECULE: DOWN SYNDROME CELL ADHESION MOLECULES;
26:	3moq-A	11.4	2.0	94	126	17	PDB	MOLECULE: NEW ANTIGEN RECEPTOR VARIABLE DOMAIN,P3(40) PEPTI
27:	6c6m-A	11.3	1.7	87	95	14	PDB	MOLECULE: MYOSIN LIGHT CHAIN KINASE, SMOOTH MUSCLE;
28:	2wwk-O	11.3	2.0	92	99	13	PDB	MOLECULE: TITIN;
29:	1ya5-A	11.2	14.2	126	198	17	PDB	MOLECULE: N2B-TITIN ISOFORM;
30:	1qz1-A	11.1	9.1	111	288	16	PDB	MOLECULE: NEURAL CELL ADHESION MOLECULE 1, 140 KDA ISOFORM;

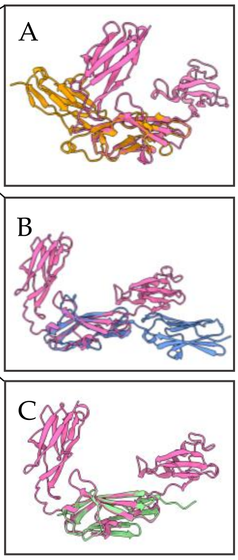

BLAST results against gp17 amino acid sequence

PHET2YK6016-Alignment-Descriptions

Description	Scientific Name	Max Score	Total Score	Query Cover	E value	Per. ident	Acc. Len	Accession
**Hoc-like head decoration [Escherichia phage N4]**	Escherichia phage N4	557	557	100%	0.0	100.00	279	YP_950495.1
**fibritin neck whisker protein [Escherichia phage AlfredRasser]**	Escherichia phage AlfredRasser	556	556	100%	0.0	100.00	290	QXV75832.1
**32 kDa protein [Escherichia phage N4] [Escherichia phage vB_Eco_SPSP]**	Escherichia phage vB_Eco_SPSP	555	555	100%	0.0	99.64	279	CAH0462260.1
**hypothetical protein AC3HA13_170 [Escherichia phage vB_EcoP_3HA13]**	Escherichia phage vB_EcoP_3HA13	555	555	100%	0.0	99.64	279	QDF14915.1
**hypothetical protein vec25_17 [Escherichia phage VEc25]**	Escherichia phage VEc25	555	555	100%	0.0	99.28	279	QPN96283.1
**capsid decorating protein [Escherichia phage OLB145]**	Escherichia phage OLB145	554	554	100%	0.0	99.28	279	AYR04200.1
**neck whiskers protein [Escherichia phage PMBT57]**	Escherichia phage PMBT57	547	547	100%	0.0	98.57	279	AUV59093.1
**Hoc-like head decoration [Escherichia phage Bp4]**	Escherichia phage Bp4	392	392	100%	9E-135	71.79	277	YP_009032039.1
**Hoc-like head decoration [Escherichia phage ECBP1]**	Escherichia phage ECBP1	389	389	100%	2E-133	72.14	277	YP_006908790.1
**capsid decorating protein [Escherichia phage vB_EcoS_Uz-1]**	Escherichia phage vB_EcoS_Uz-1	387	387	100%	1E-132	70.36	277	UWJ04367.1
**Hoc-like head decoration [Shigella phage pSb-1]**	Shigella phage pSb-1	387	387	100%	2E-132	71.07	277	YP_009008459.1
**fibritin (wac) protein [Escherichia phage VTCCBPA_322]**	Escherichia phage VTCCBPA_322	385	385	100%	6E-132	70.36	277	XFC51964.1
**hypothetical protein DPIBCGCG_00062 [Escherichia phage KKP 3715]**	Escherichia phage KKP 3715	384	384	100%	1E-131	68.57	277	WLW40976.1
**fibritin protein [Escherichia phage vB_EcoM_PD205]**	Escherichia phage vB_EcoM_PD205	383	383	100%	7E-131	69.64	292	UVK80559.1
**hypothetical protein [Caudoviricetes sp.]**	Caudoviricetes sp.	381	381	100%	3E-130	68.93	277	QHJ78181.1
**Hoc-like head decoration [Escherichia phage vB_EcoP_SP5M]**	Escherichia phage vB_EcoP_SP5M	380	380	99%	1E-129	69.06	277	YP_010659531.1
**Hoc-like head decoration [Escherichia phage vB_EcoP-ZQ2]**	Escherichia phage vB_EcoP-ZQ2	380	380	100%	1E-129	69.64	277	YP_010660018.1
**Hoc-like head decoration [Escherichia phage IME11]**	Escherichia phage IME11	379	379	100%	2E-129	69.29	277	YP_006990661.1
**Hoc-like head decoration [Escherichia phage vB_EcoP_PhAPEC7]**	Escherichia phage vB_EcoP_PhAPEC7	379	379	100%	2E-129	67.50	277	YP_009056149.1
**Hoc head outer capsid protein [Escherichia phage UE-S5a]**	Escherichia phage UE-S5a	377	377	100%	1E-128	68.93	277	WVP99895.1
**Hoc-like head decoration [Escherichia phage St11Ph5]**	Escherichia phage St11Ph5	377	377	100%	2E-128	69.29	277	YP_010659873.1
**fibritin (wac) protein [Escherichia phage UE-S5b]**	Escherichia phage UE-S5b	376	376	100%	2E-128	68.57	277	WWE95469.1
**immunoglobulin domain-containing protein [Escherichia coli]**	Escherichia coli	375	375	100%	5E-128	68.93	277	WP_241362664.1
**Hoc-like head decoration [Escherichia phage EC1-UPM]**	Escherichia phage EC1-UPM	375	375	100%	9E-128	69.29	277	YP_009598292.1
**structural protein [Escherichia phage E20]**	Escherichia phage E20	375	375	100%	9E-128	67.50	277	WAQ79278.1
**Hoc-like head decoration [Escherichia phage vB_EcoP_PhAPEC5]**	Escherichia phage vB_EcoP_PhAPEC5	375	375	100%	1E-127	67.50	277	YP_009055525.1
**Hoc-like head decoration [Escherichia phage U1G]**	Escherichia phage U1G	374	374	100%	2E-127	68.93	277	YP_010659777.1
**hypothetical protein EI543_13605 [Enterococcus faecium]**	Enterococcus faecium	373	373	100%	6E-127	68.57	277	QHQ49142.1
**Hoc-like head decoration [Escherichia phage PGN829.1]**	Escherichia phage PGN829.1	372	372	100%	1E-126	68.57	286	YP_010659694.1
**fibritin protein [Escherichia phage ST4]**	Escherichia phage ST4	372	372	100%	1E-126	68.57	277	UUB18099.1
**TPA: hypothetical protein [Bacteriophage sp.]**	Bacteriophage sp.	374	374	100%	1E-126	68.57	320	DAE39532.1

Supplementary Figure S7. Structural comparison of IG-like domains via DALI search. *A*. Results of the closest 50 search results from DALI server against PDB of N4 gp17a. The top two search results (*B*, *C*) were overlayed in UCSF ChimeraX and an RMSD for pruned atoms (only regions which overlay) was calculated for each. The aa sequence of N4 gp17 was also submitted to BLAST, which returned sequence matches to N4 itself as well as a large array of coliphages. All high-fidelity matches belonged to coliphages, with the exception of one *Shigella* phage sharing high sequence homology at 71%, and an *Enterococcus* phage at 69%. Coliphage AlfredRasser has a 100% identical protein identified as a neck whisker protein.

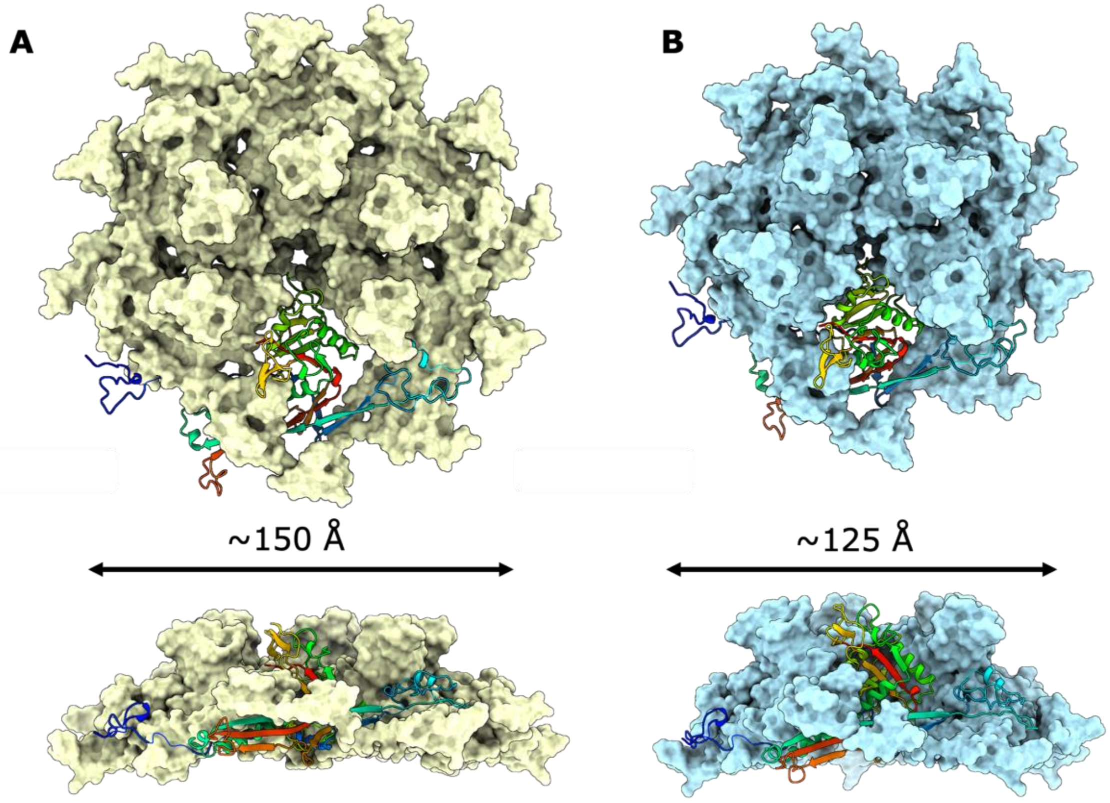

Supplementary Figure S8. MCP chains in context of capsomers. (**A**) Hexamer 1 shown in axial and side view, with chain B in ribbon representation (Jones' rainbow) and chains C-G in surface representation, visualised in UCSF ChimeraX. The molecular surface is calculated based on the PDB file in UCSF ChimeraX represented as a smoothed van-der Waals surface. (**B**) Pentamer shown as per panel A, with five copies of MCP chain A.

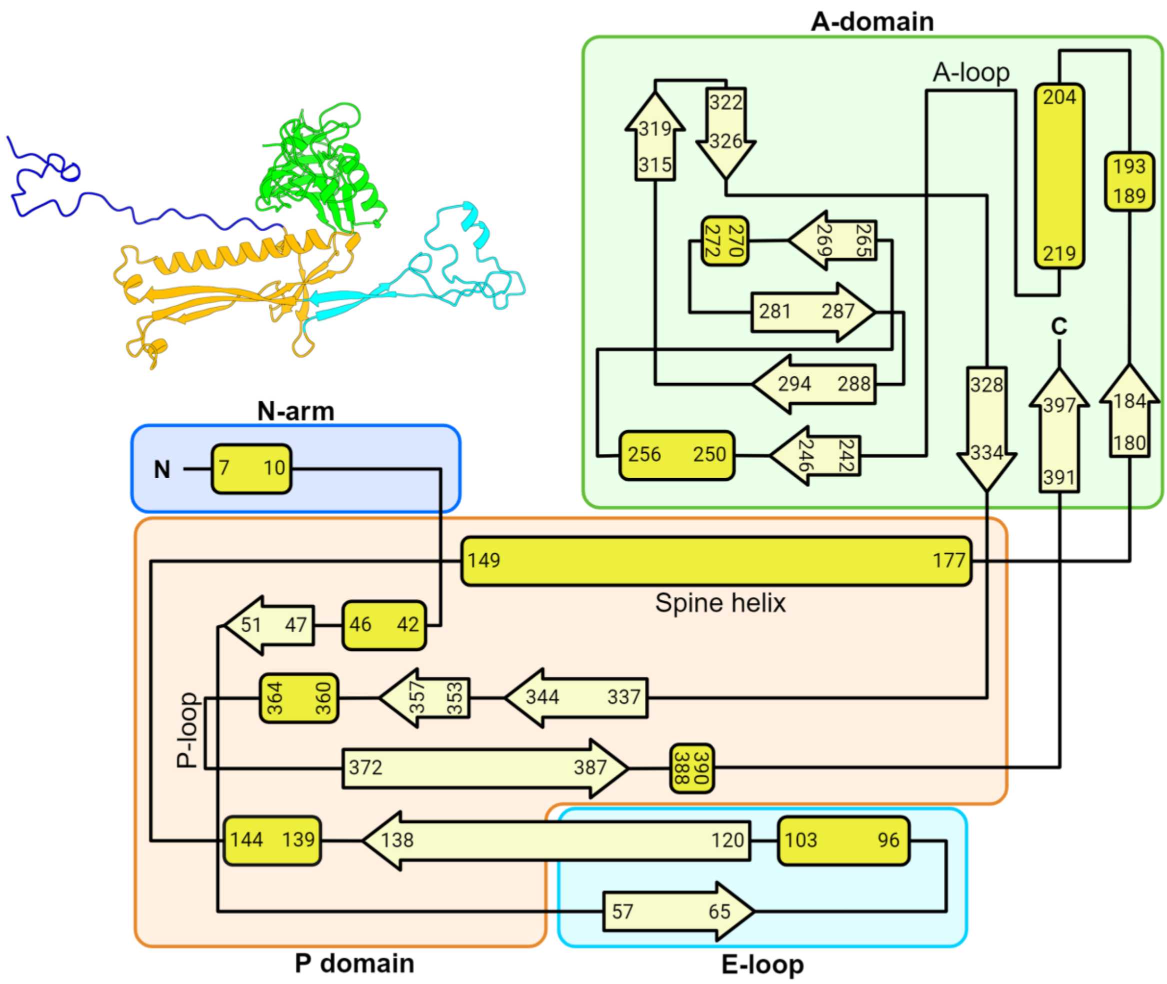

Supplementary Figure S9. MCP topology diagram. Topology diagram replotted manually from results generated using PDBSum for visual interpretability. Major conserved motifs of the HK-97 fold are highlighted and coloured accordingly in the inset ribbon representation of [41] chain I. Selected secondary structure features are labelled.

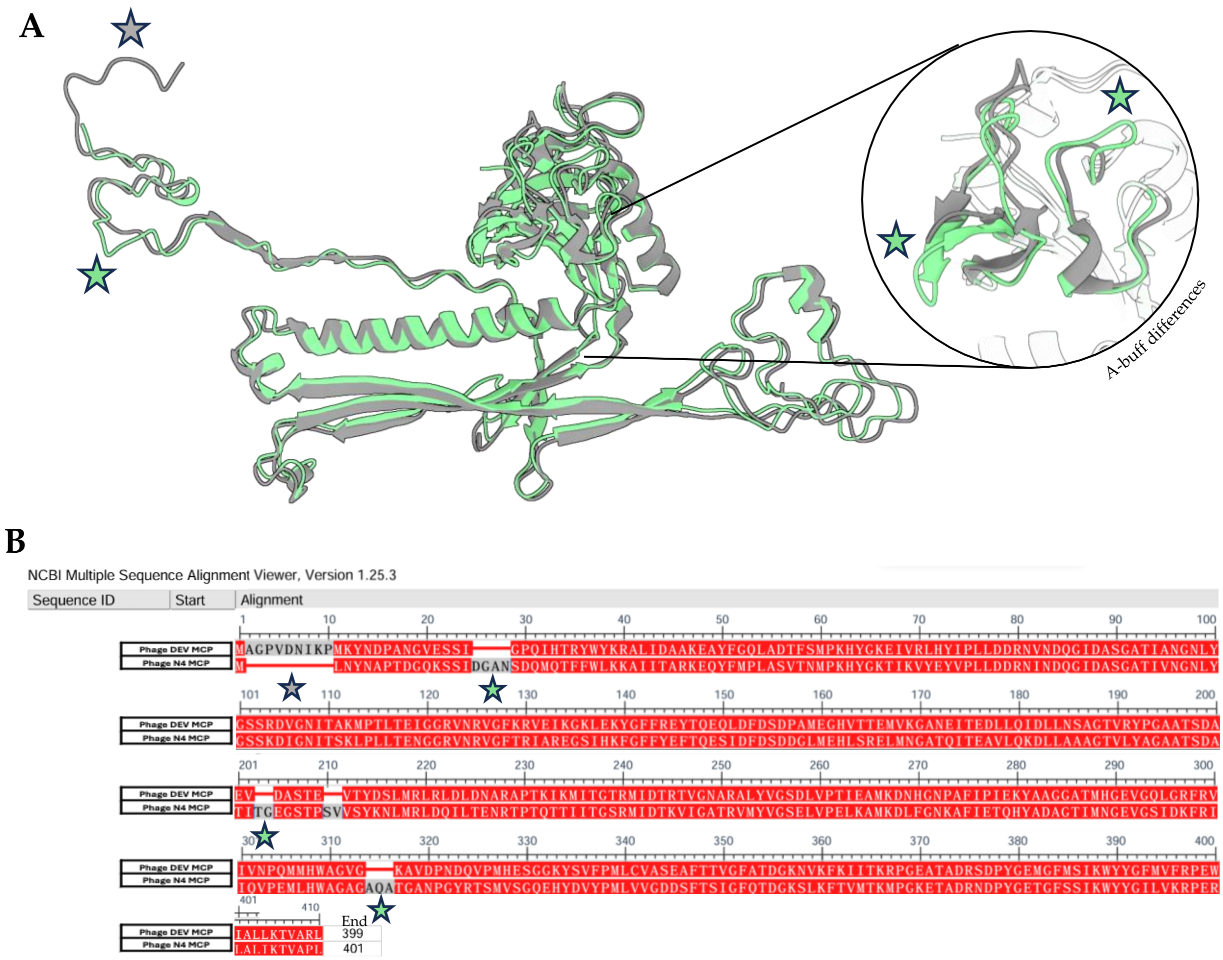

Supplementary Figure S10. Comparison of the DEV and N4 major capsid proteins (A) Overlay of the MCP structures of N4 chain I (green) and the equivalent chain in the DEV capsid (pdb 9BGN, chain I, coloured grey), with an overall RMSD of 1.9 Å. Sites where additions are present in a given chain when compared with the other are marked by stars of the corresponding colour, including for the A-buff inset. (B) Sequence alignment of N4 and DEV MCP amino acid sequences performed using the NCBI Constraint-based Multiple Alignment Tool (COBALT) [35]
